# Mitigation co-benefits of carbon sequestration from MGNREGS in India

**DOI:** 10.1371/journal.pone.0251825

**Published:** 2021-05-20

**Authors:** N. H. Ravindranath, Indu K. Murthy

**Affiliations:** Centre for Sustainbale Technologies, Indian Institute of Science, Bangalore, India; University of California Davis, UNITED STATES

## Abstract

Mahatma Gandhi Rural Employment Guarantee Scheme a large social security programme being implemented in India, with an average annual investment of US$ 7 billion. The bulk of the activities under this programme are focused on natural resources such as land, water and trees, which provide adaptation benefits. In this study an attempt is made to estimate the carbon sequestration achieved and future potential, as a co-benefit, from MGNREGS. The total mean carbon sequestered at the national level, considering the cumulative number of natural resource based activities, for the year 2017–18 was estimated to be 102 MtCO_2_. The annual mean carbon sequestration is projected to increase to about 132 MtCO_2_ by 2020 and 249 MtCO_2_ by 2030. Drought proofing is one of the activities implemented under MGNREGS and it includes tree planting, relevant to achieving the NDC carbon sink target. The cumulative carbon sink created by drought proofing activities is projected to be 56 MtCO_2_ in 2020, 281 MtCO_2_ in 2025 and 561 MtCO_2_ in 2030. This study demonstrates the significant carbon sink potential of MGNREGS and highlights the importance of estimation and reporting climate mitigation co-benefits of adaptation actions such as MGNREGS under the Paris Agreement.

## 1. Introduction

The Mahatma Gandhi National Rural Employment Guarantee Scheme (MGNREGS), is a social security programme, launched in 2006 with an objective to ensure livelihood security of rural people by guaranteeing at least 100 days of wage employment in a financial year, to every household, whose adult members volunteer to work. MGNREGS is the largest wage employment programme in the world. According to MGNREGS Guidelines, “Planned and systematic development of land and harnessing of rainwater following watershed principles are the central focus to sustainably enhance farm productivity and income of poor people”. Therefore, activities implemented under MGNREGS are largely focused on improving land and water resources. These activities (referred to as ‘Works’ under MGNREGS) generate environmental benefits such as increased irrigation provisioning, ground water recharge, soil, water and biodiversity conservation, increased food production, halting land degradation and building resilience to current climate risks such as moisture stress, delayed rainfall, droughts and floods [1–3]. Apart from reducing vulnerability to climate variability and change [2], MGNREGS-NRM (Natural Resource Management) activities have the potential to sequester carbon in soil and biomass under different activities such as land development, soil and water conservation, enhanced irrigation and tree planting activities—leading to increased tree growth, crop biomass production and soil carbon enhancement. Limited evidence is available on the actual or potential impact of MGNREGS on carbon sequestration for mitigation of climate change.

Given the scale of the programme, with an average annual investment of US$ 7 billion, and a focus on NRM, it is necessary to assess the climate change ‘mitigation co-benefits’ of the programme. The present study aims to assess the carbon sequestration co-benefits of MGNREGS and its future potential to deliver mitigation co-benefits. Further, the contribution of mitigation co-benefits of MGNREGS works in meeting India’s Nationnaly Determined Contribution (NDC) target of 2.5 to 3 GtCO_2_ of additional CO_2_ sequestration by 2030 is estimated.

## 2. Material and methods

The broad approach to estimating the mitigation co-benefits of MGNREGS involved adopting the Agro-Ecological Region (AER) stratification, identifying sample districts, blocks and villages, conducting field studies for estimating carbon and average area implemented per NRM activity, compilation of data from the MGNREGS database to obtain cumulative number of activities implemented under each activity upto 2017–18, and finally projecting carbon stocks under each of the NRM activities to 2030.

### 2.1 Stratification

AER (Agro Ecological Region) approach is adopted to stratify India. This stratification is adopted by agricultural universities, agriculture departments and other development programmes. In this study, 18 AERs excluding AER 1 (Western Himalayas, Ladakh Plateau and north Kashmir), and AER 20 (A&N and Lakshadweep islands) were selected.

#### 2.1.1 Selection of districts in AERs

This required aggregating the geographich area of all districts in every AER, and selection of districts for sample studies. Considering the resources and time available, 32 districts, accounting for about 5% of the total number of districts in India were selected. The number of districts selected per AER was proportional to its percentage share in area to total area of all the AERs.

#### 2.1.2 Selection of blocks in a district

A district consists of blocks. Sample blocks in the identified districts were selected considering the mean number of activities implemented per block in a district, arranging the blocks in ascending order based on number of activities, and selecting two blocks nearest to mean number of activities implemented per block for each selected sample district.

#### 2.1.3 Selection of villages in a block

The final unit of sampling for estimating carbon sequestration potential of MGNREGS activities is a village. Three villages were selected per block based on the population of the villages (small, medium and large).

### 2.2 Methods for estimating biomass and soil carbon stocks

The methods for estimating biomass and soil organic carbon stocks and the calculation methods for obtaining carbon sequestration or stock change estimates are:

**Step 1: Selection of MGNREGS activities in a village**
  - Through Participatory Rural Appraisal, all MGNREGS-NRM activities implemented in a village were identified.
  - Only those activities carried out prior to 2014–15, i.e. upto 2013–14 were included in the study, since it is possible to measure the biomass and soil carbon impacts, only after a minimum of 3-years after implementation of an activity.
**Step 2: Stratification of MGNREGS activities and selection of carbon pools**
  MGNREGS activities could be grouped into two categories, namely activities involving tree planting, with implications for both biomass and soil carbon and those involving no tree planting, but with implications for soil carbon.
**Step 3: Selection of methods for estimation of carbon sequestration**
  Carbon sequestration from implementation of MGNREGS activities is estimated by sampling in two types of plots:
  1. MGNREGS-NRM activity implemented plots—for estimating biomass and/or soil carbon pools
  2. Control plots—for comparison and assessment of change or impact of MGNREGS-NRM activities.
  The difference in carbon stocks of ‘MGNREGS impacted plots’ and ‘control plots’ is used to estimate the carbon sequestration or stock change. Calculation of the annual rate of sequestration per ha per year is based on the number of years post-implementation of the activity (t/ha/year).
**Step 4: Biomass (carbon) estimation**
  - Above ground biomass consists of trees and shrubs. Standard plot method [4, 5] was adopted.
    - 3 to 5 plots of 25 x 25 meters were marked randomly in the field
    - Diameter at Breast Hight (DBH) was measured and height estimated.
  - Biomass above ground in trees was estimated using biomass equations by Ravindranath et al. [4]
**Step 5: Soil carbon estimation**
  Soil organic carbon (SOC) was estimated for both tree-based as well as non-tree based MGNREGS-NRM activities. Plot selection and soil sample collection for laboratory estimation was done following Ravindranath et al. [4].
  - Soil samples collected from 3 to 5 plots marked for biomass estimation or soil samples collected for each activity (in case of non-tree based activities) from farms (if large farm size-5 plots, small farm size–3 plots)
**Step 6: Estimation of carbon sequestration for each MGNREGS-NRM activity**
  - Biomass and soil carbon sequestration or stock change was estimated for each activity in tC/ha/year, considering stocks estimated for ‘MGNREGS-impacted’ and ‘control plots’.
  - Rate of change per annum was estimated taking into consideration the number of years post-implemtation of an activity.

## 3. Results

Carbon sequestration from MGNREGS-NRM activities was estimated using information on:

- Cumulative number of activities implemented upto 2017–18
- Mean number of activities implemented during 2013–14 to 2017–18
- Number of activities implemented projected for 2020, 2025 and 2030, considering mean annual rates of implementation during 2013–14 to 2017–18
- Average area impacted by each NRM activity, derived from field studies in sample villages
- Average carbon sequestration rates in tC/ha/year (biomass and SOC) for different activities in different districts in different AERs.

### 3.1 Estimation of cumulative number of activities implemented upto 2017–18

The MGNREGS database provides data on number of activities implemented annually. The cumulative number of NRM activities leading to carbon sequestration was estimated at the AER level using this database. The cumulative number of major MGNREGS-NRM activities with potential to contribute to carbon sequestration is given AER-wise in Table 1.

**Table 1:** Cumulative number of major NRM activities implemented under MGNREGS upto 2017–18.

AERs	Drought proofing	Micro irrigation	Renivation of traitional water bodies	Land development	Water conservation & harvesting
AER2	34528	321424	197351	384995	216663
AER3	271521	435259	1318398	5454090	1759371
AER4	145561	187674	107241	301717	126431
AER5	184285	261933	54018	934664	196027
AER6	26076	160982	1450	29286	30702
AER7	5804	462557	28500	35237	108426
AER8	138426	378112	40264	307511	269470
AER9	305656	394655	195069	725981	295968
AER10	35929	139294	14111	291086	45217
AER11	7699	176076	155980	419420	90443
AER12	141231	281017	45337	386205	270761
AER13	63832	251536	267858	306296	705931
AER14	1098865	230729	722837	1396372	319548
AER15	27253	157633	82298	173280	151343
AER16	5304	222733	78867	189713	139400
AER17	25206	202316	75540	248403	101372
AER18	186220	296324	192363	176298	312604
AER19	81175	264572	99590	755636	161916

### 3.2 Projection of number of MGNREGS-NRM activities upto 2030

Assuming MGNREGS will continue to be implemented upto 2030, based on the fact that the annual investment has increased over the years since its inception [6], the following approach was adopted for projecting the number of activities upto 2030.

- Estimate the average annual rate of implementation of each MGNREGS-NRM activity, based on data from MGNREGS database for the period of 2014 to 2018 (Table 2).
- Using the annual mean rate of implementation of each NRM activity for the period 2014 to 2018, project the number of activities implemented upto 2027 (as activities implemented after 2027 may provide carbon benefit only beyond 2030, and therefore considered not relevantfor this study), at constant rates.

**Table 2:** Cumulative, annual and mean number of major NRM activities implemented for the period 2006–07 to 2017–2018 and projections upto 2030.

Major NRM activities	Cumulative number of activities implemented up to 2017–18	Mean number and CV (%) of activities during 2013–14 to 2017–18	Projection of cumulative number of NRM activities to be implemented during		
			2020	2025	2027
Drought proofing	4824827	272389 (10.5%)	2909604	4271546	5633489
Micro irrigation	3677073	181129 (2.7%)	1977004	2882651	3788299
Water conservation & harvesting	8081482	500502 (3.7%)	7642551	10145059	12647566
Renovation of traditional water bodies	5301592	235695 (9.1%)	2755877	3861706	4967534
Land development	12516189	628388 (6.6%)	6335448	9477389	12619331

*Projections for 2020, 2025 and 2027 (2030) assume linear increase in the mean number of activities implemented during 2013 to 2018.

### 3.3 Estimation of average area implemented under each MGNREGS-NRM activity in different AERs

The basic data required for estimating carbon sequestration is—area subjected to each MGNREGS-NRM activity in each village, extrapolated to the national level. The MGNREGS Database does not provide this data. Thus in this study, area subjected to different NRM activities in the sample villages was estimated through survey and field measurements. Since the area impacted for a given NRM activity, say minor irrigation or drought proofing may vary among AERs, measurements were conducted in different samples villages across AERs (Table 3). For a majority of the activities, the area impacted per activity was <2 ha.

**Table 3:** Average area impacted by MGNREGS-NRM activities in different AERs and average biomass and soil carbon sequestration rates (tC/ha/yr) for each activity.

AERs	Number of sampled		MGNREGS activities	Average area*/ activity (ha)	Carbon** (tC/ha/year)		
	Districts	Villages			Soil	Biomass	Total
AER2	4	22	Micro irrigation activities	0.90	1.10		1.10
				0.66	-1.46		-1.46
					0.02		0.02
			Land development	2.28	1.05		1.05
					1.97		1.97
			Drought proofing	1.15	2.07	2.20	4.27
AER3	1	4	Water conservation and harvesting	7.25	-0.85		-0.85
				0.71	0.88		0.88
			Land development	2.28	1.37		1.37
			Drought proofing	1.15	2.61	1.89	4.50
			Water conservation and harvesting	0.99	-1.05		-1.05
AER4	3	18	Minor irrigation activities	0.66	0.20		0.20
					0.35		0.35
			Land development	2.28	-0.90		-0.90
			Water conservation and harvesting	0.71	0.73		0.73
				0.99	-0.51		-0.51
				7.25	0.65		0.65
			Drought proofing	0.75	0.70	0.95	1.65
AER5	2	12	Water conservation and harvesting	0.71	0.73		0.73
				0.99	0.95		0.95
			Land development	2.28	1.06		1.06
					-0.88		-0.88
			Drought proofing	1.80	0.56	1.05	1.61
			Minor irrigation activities	0.66	-0.66		-0.66
					0.08		0.08
			Water conservation and harvesting	0.71	1.64		1.64
AER6	4	24	Drought proofing	1.15	-0.21	1.13	0.92
			Renovation of traditional water bodies including desilting of tanks	0.90	1.37		1.37
			Land development	2.28	-0.02		-0.02
			Minor irrigation activities	0.66	0.33		0.33
					0.36		0.36
			Water conservation and harvesting	7.25	0.33		0.33
AER7	1	6	Minor irrigation activities	0.66	1.93		1.93
					-0.23		-0.23
			Drought proofing	0.78	1.23	2.2	3.43
			Minor irrigation activities				
AER8	2	9	Water conservation and harvesting	0.71	-0.13		-0.13
			Land development	2.28	0.10		0.10
			Drought proofing	0.78	0.12	1.16	1.28
				0.66	-0.97		-0.97
AER9	1	4	Renovation of traditional water bodies including desilting of tanks	0.90	0.78		1.21
AER10	2	12	Minor irrigation activities	0.66	-0.61		-0.61
			Drought proofing	1.82	0.83	1.15	1.98
			Water conservation and harvesting	0.71	1.19		1.19
			Land development	2.28	0.28		0.28
AER11	1	5	Water conservation and harvesting	0.71	0.44		0.44
				0.99	0.22		0.22
			Land development	2.28	-0.07		-0.07
			Drought proofing	1.95	0.96	0.98	1.94
			Minor irrigation activities	0.66	1.27		1.27
AER12	2	8	Land development	2.28	0.29		0.29
			Drought proofing	1.15	0.70	1.35	2.05
			Water conservation and harvesting	7.25	0.36		0.36
AER13	1	4	Water conservation and harvesting	0.71	1.90		1.90
			Drought proofing	2.30	2.24	1.15	3.39
			Minor irrigation activities	0.66	0.70		0.70
AER14	2	8	Minor irrigation activities	0.66	1.43		1.43
					0.88		0.88
			Land development	2.28	1.15		1.15
			Drought proofing	1.10	-0.68	0.97	0.29
AER15	1	4	Water conservation and harvesting	0.71	-1.73		-1.73
			Drought proofing	0.90	0.55	2.1	2.65
			Land development	2.28	-0.01		-0.01
			Minor irrigation activities	0.66	-1.08		-1.08
AER16	1	4	Water conservation and harvesting	0.71	-0.20		-0.20
			Minor irrigation activities	0.66	-1.97		-1.97
					-0.30		-0.30
			Drought proofing	1.19	0.93	1.18	2.11
AER17	1	4	Land development	2.28	0.12		0.12
			Drought proofing	1.10	0.14	0.95	1.09
			Minor irrigation activities	0.66	-0.38		-0.38
					0.21		0.21
AER18	1	6	Renovation of traditional water bodies including desilting of tanks	0.90	0.73		0.73
			Drought proofing	1.37	0.87	1.15	2.02
			Water conservation and harvesting	0.71	0.19		0.19
			Minor irrigation activities	0.66	0.40		0.40
AER19	1	4	Drought proofing	1.10	1.07	0.85	1.92
			Water conservation and harvesting	0.71	1.72		1.72
			Minor irrigation activities	0.66	0.54		0.54
			Land development	2.28	-0.10		-0.10

*The average area impacted for different AERs is estimated based on the activity implemented in the sample villages. In some AERs even though a activity is implemented, the sample villages did not contain that NRM activity. In such cases, the average area value for a given NRM activity is obtained from the neighbouring district/AER

** It is normal to obtain negative carbon sequestration rates, especially in agricultural lands due to variations in factors such as cultivation practices (ploughing and inter-culture operations), application of organic manure, incorporation of crop residue into soil or removal of the residue from the crop fields, soil slope, etc.

### 3.4 Carbon sequestration rates for MGNREGS-NRM activities

Carbon sequestration rates (tC/ha/yr) for each NRM-based activity was calculated and extrapolated to village, district and AER scales. Biomass sequestration rates were estimated only for drought proofing activity. SOC was estimated for all activities involving tree planting and no tree planting such as land development, minor irrigation activities, water conservation and water harvesting.

The carbon sequestration rates varied for a given NRM activity across AERs (Table 3). The carbon sequestration rates for drought proofing ranged from 0.85 to 2.20 tC/ha/yr for biomass carbon and 0.12 to 2.61 tC/ha/yr for SOC. The carbon sequestration rates for land development was estimated to be in the range of 0.1 to 1.97 tC/ha/yr for SOC and for minor irrigation activities (0.1 to 1.93 tC/ha/yr). The carbon sequestration rates were positive for most of the NRM activities in a majority of the AERs. However, negative carbon sequestration rates for SOC, were recorded for some activities in some of the AERs as carbon is being released from the soils as a result of the activities being implemented, crop production practices, soil slope and other factors.

### 3.5 Total carbon sequestration through MGNREGS-NRM activities during 2017–18

The main aim of this study was to estimate annual aggregate carbon sequestration achieved by MGNREGS and its contribution to mitigation of climate change at the national level for India. Carbon sequestration estimates for each AER and national level aggregate considering all AERs are given in Table 4.

**Table 4:** Total carbon (MtC and MtCO_2_) sequestered by MGNREGS-NRM activities during 2017–18.

AERs	Total carbon sequestered by different NRM activities in 2017–18 (MtC)						Total sequestration during 2017–18 (MtCO_2_)
	Land development	Micro irrigation	Water conservation and harvesting	Renovation of traditional water bodies	Drought proofing	Total of all activities	
AER2	1.865	-0.002	0.647	0.362	0.791	3.663	13.43
AER3	3.414	-0.010	-1.808	0.425	0.681	2.702	9.91
AER4	0.201	0.412	0.152	0.150	0.137	1.052	3.86
AER5	-0.139	-0.007	0.891	1.288	0.109	2.142	7.85
AER6	-0.016	0.193	0.503	0.370	0.045	1.095	4.02
AER7	0.715	0.190	0.223	0.438	0.060	1.626	5.96
AER8	-0.212	0.109	-0.192	0.072	0.116	-0.107	-0.39
AER9	0.187	0.053	0.136	0.169	0.059	0.604	2.21
AER10	0.153	-0.121	0.210	0.030	0.510	0.782	2.87
AER11	0.029	0.071	0.061	0.008	1.193	1.362	4.99
AER12	0.588	0.527	0.682	0.337	0.160	2.294	8.41
AER13	-0.010	1.149	1.729	0.925	1.107	4.900	17.97
AER14	0.189	0.299	0.470	0.109	0.013	1.080	3.96
AER15	0.057	-0.050	0.002	0.026	0.817	0.852	3.12
AER16	-0.101	-0.009	-0.023	0.283	0.648	0.798	2.93
AER17	0.449	-0.002	-0.047	0.583	0.328	1.311	4.81
AER18	-0.005	0.299	0.259	0.007	0.053	0.613	2.25
AER19	-0.018	0.561	0.313	0.086	0.119	1.061	3.89
**Total**	**7.35**	**3.66**	**4.21**	**5.67**	**6.95**	**27.83**	**102.04**

It can be observed that carbon sequestration is positive for a majority of activities in a majority of the AERs. The total carbon (biomass and SOC) sequestered at the national level, covering all AERs and all the MGNREGS-NRM activities, for the year 2017–18 (considering cumulative activities implemented) is estimated to be 16.9 MtC (61.9 MtCO_2_).

#### 3.5.1 Biomass and soil carbon sequestration (MtC) by drought proofing activities

Drought proofing activities include tree planting through afforestation and horticultural fruit tree planting. Tree biomass and SOC estimates were made separately and the same is presented in Table 5. It is seen that biomass carbon sequestration accounted for 3.84 MtC and SOC for 3.04 MtC. Overall, Drought proofing activity accounted for a little over 40% of the total carbon sequestration by all NRM activities, implemented under MGNREGS, at the national level.

**Table 5:** Biomass and SOC sequestration (MtC and MtCO_2_) by drought proofing activities during 2017–18.

	Carbon sequestered in biomass (MtC)	Carbon sequestered in soil (MtC)	Total carbon sequestered in biomass and soil (MtC)
AER2	0.407	0.383	0.791
AER3	0.286	0.395	0.681
AER4	0.079	0.058	0.137
AER5	0.071	0.038	0.109
AER6	0.056	-0.010	0.045
AER7	0.038	0.021	0.060
AER8	0.105	0.011	0.116
AER9	0.000	0.059	0.059
AER10	0.296	0.214	0.510
AER11	0.602	0.590	1.193
AER12	0.105	0.054	0.160
AER13	0.375	0.732	1.107
AER14	0.044	-0.031	0.013
AER15	0.647	0.170	0.817
AER16	0.362	0.286	0.648
AER17	0.287	0.041	0.328
AER18	0.030	0.023	0.053
AER19	0.052	0.066	0.119
**Total in MtC**	**3.84**	**3.04**	**6.95**
**Total in Mt CO**_**2**_	**14.08**	**11.15**	**25.48**

### 3.6 Carbon sequestration projections from 2017 to 2030 for India

In Section 3.5, carbon sequestration was estimated for MGNREGS-NRM activities implemented upto 2017–18. Carbon sequestration projected for the period 2020 to 2030 shows a continuous increase (Table 6), due to increase in the cumulative number of NRM activities implemented under MGNREGS.

**Table 6:** Projections of annual net carbon sequestration by AER in 2020, 2025 and 2030 (MtCO_2_) for India.

AERs	Total carbon sequestration by MGNREGS-NRM activities during 2020 (MtCO_2_)	Total carbon sequestration by MGNREGS-NRM activities during 2025 (MtCO_2_)	Total carbon sequestration by MGNREGS-NRM activities during 2030 (MtCO_2_)
AER2	15.26	21.98	29.14
AER3	10.18	15.92	20.13
AER4	4.96	7.22	9.88
AER5	9.24	12.03	14.09
AER6	6.31	8.02	10.18
AER7	8.50	10.03	11.89
AER8	4.87	6.59	8.74
AER9	4.41	5.75	7.71
AER10	3.72	5.65	8.00
AER11	6.33	11.56	17.24
AER12	10.46	13.42	16.86
AER13	21.45	26.20	34.68
AER14	5.51	8.10	10.69
AER15	3.64	5.55	8.74
AER16	3.82	5.74	8.60
AER17	5.30	10.97	16.83
AER18	3.06	4.45	6.32
AER19	5.00	6.81	9.28
**Total**	**132.00**	**186.00**	**249.00**

During 2017, total carbon sequestered was estimated to be 102 MtCO_2_. The annual quantity of carbon sequestration is projected to increase in different years upto 249 MtCO_2_ by 2030 (estimated likely range is 150 to 540 MtCO_2_). Thus, even though MGNREGS is a livelihood security programme, the carbon sequestration co-benefits of the programme are significant.

## 4. Discussion

According to IPCC [7], most categories of adaptation options for climate change in land use sectors have positive impacts on mitigation. Similarly, mitigation choices taken in a particular land-use sector may enhance or reduce resilience to climate variability and change within or across sectors. Smith and Olesen [8] and Ravindranath [9] have identified a number of synergies between mitigation options in agriculture and forestry, which also enhance resilience to climate change.

**Fig 1:**
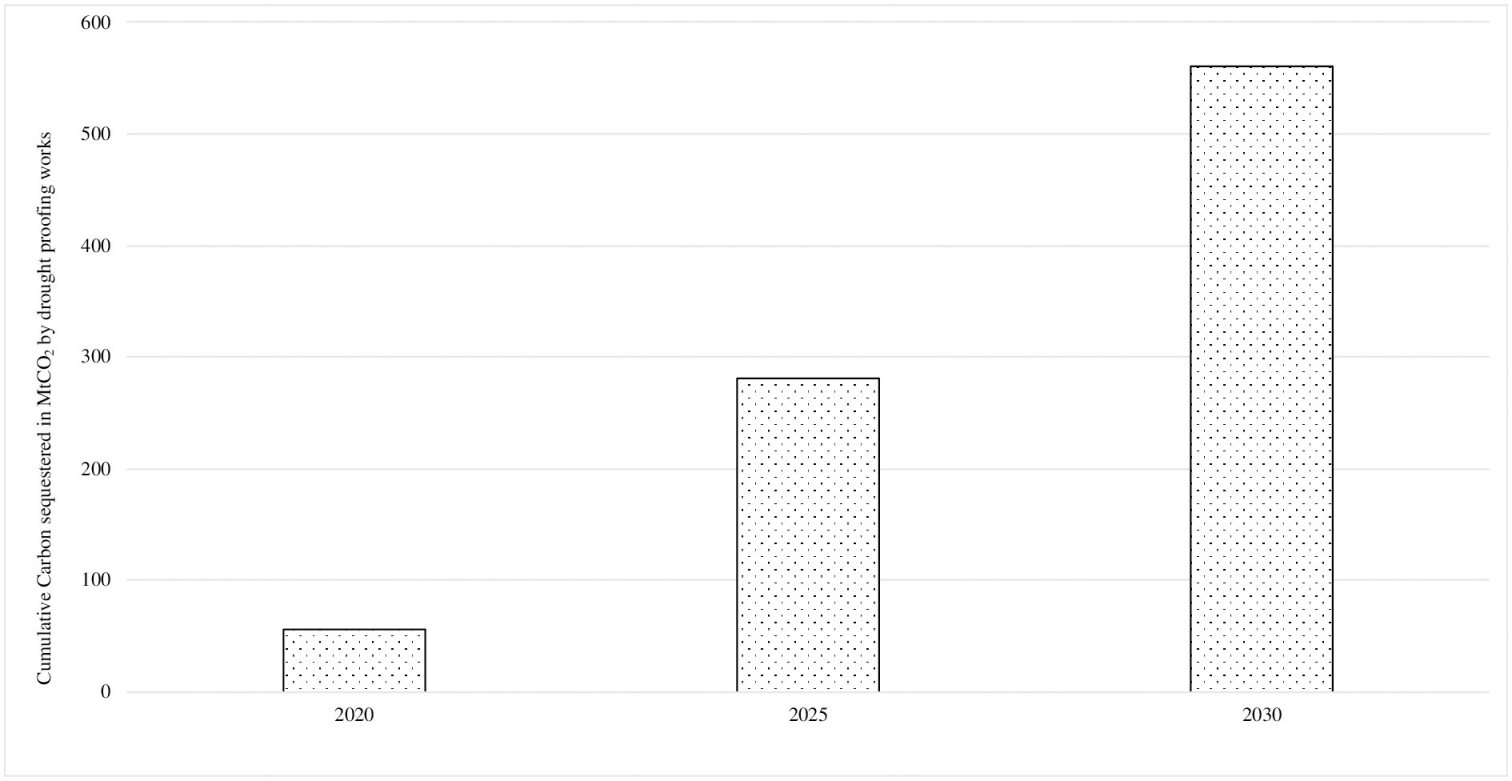
Cumulative carbon sequestration under drought proofing works of MGNREGS.

One of the targets of India’s NDC is to “create an additional carbon sink of 2.5 to 3.0 billion tonnes of CO_2_-equivalent through additional forest and tree cover by 2030” [10]. The NDC target includes only lands subjected to enhancing forest and tree cover through tree planting. Thus, only drought proofing activity, which includes tree planting would qualify for meeting the carbon sink target of the NDC. Drought proofing activity is projected to increase from 56 MtCO_2_ in 2020 to 281 MtCO_2_ in 2025 and 561 MtCO_2_ in 2030 (Fig 1). This is quite significant in comparison to the total CO_2_ sequestration of 308 MtCO_2_, estimated for forest, agriculture, settlements and other land categories for 2016, according to the Third Biennial Update Report [11].

This clearly establishes that NRM activities implemented under MGNREGS can make significant contribution to climate change mitigation and contribute to meeting the NDC forestry target of the Government of India. This becomes all the more important when it is unlikely that forest sector alone may not be able to meet the NDC carbon sink target of 2.5 to 3 billion tonnes of CO_2_ by 2030. There is therefore a need to incorporate tree planting, especially fruit and fodder yielding trees into most NRM activities, under MGNREGS, with an aim of generating alternate income and livelihood sources from trees, while carbon sequestration will be a co-benefit. This would require periodic monitoring and reporting of the carbon sequestration benefits, which is also a requirement for Adaptation Communciation under Article 7 of the Paris Agreement.

The current study demonstrated the potential of MGNREGS to contribute to mitigation goals and targets. However, these estimates are preliminary as i) the sample size was small (due to limitations of resources and time), ii) there was absence of data on area impacted by each MGNREGS activity at a village level, iii) carbon sequestration rates across activities could vary both spatially and temporally, even within a district, iv) projections of demand for MGNREGS upto 2030 is assumed to be linear.

Given MGNREGS is a very large programme contributing to adaptation, with an annual budget of US$ 7 billion, there is a need to institutionalise periodic and scientific monitoring of carbon sequestered under MGNREGS claim benefits of carbon sequestration while reporting on adaptation under the Paris Agreement.
